# Allergens and β-Glucans in Dutch Homes and Schools: Characterizing Airborne Levels

**DOI:** 10.1371/journal.pone.0088871

**Published:** 2014-02-14

**Authors:** Esmeralda J. M. Krop, José H. Jacobs, Ingrid Sander, Monika Raulf-Heimsoth, Dick J. J. Heederik

**Affiliations:** 1 Institute for Risk Assessment Sciences, Utrecht University, Division of Environmental Epidemiology, Utrecht, The Netherlands; 2 Institute for Prevention and Occupational Medicine of the German Social Accident Insurance, Institute of the Ruhr University Bochum (IPA), Bochum, Germany; Beijing Institiute of Otolaryngology, China

## Abstract

**Background:**

Indoor air quality has an effect on respiratory health. Children are more vulnerable to a decreased indoor air quality as their lungs are still developing. We measured levels of allergens and β-(1,3)-glucans in 19 school buildings and determined whether measured levels could be reproduced. School levels were compared to those in 169 homes and the effect of building characteristics on both home and school exposure was explored.

**Methods:**

Electrostatic Dust fall Collectors were placed in school buildings for 8 weeks and in homes for 2 weeks to collect settled airborne dust. Cat, dog, and mouse allergen levels, domestic mite antigen levels and β-(1,3)-glucans were measured in the extracts from the collectors. Results were corrected for sampling duration. Using questionnaire data, relations between measured levels and building and classroom characteristics were explored.

**Results:**

In schools, exposure levels were highest in classrooms and were influenced by the socioeconomic status of the children, the season measurements were performed, moisture status of the building and pet ownership. Repeated measurements in different seasons and over the years showed significantly different levels. Home exposure was influenced by socioeconomic status, occupancy and pet ownership. Domestic mite antigen was found in higher levels in extracts from homes compared to schools while pet allergen levels were 13 times higher in schools compared to homes without pets. For mouse allergen overall levels of exposure were low but still two times higher in schools compared to homes. Levels of β-(1,3)-glucans were also approximately two times higher in schools than in homes.

**Conclusion:**

Exposure levels of several allergens and β-(1,3)-glucans in schools differ over time and are higher than in homes. For children, exposure levels measured at school could contribute to their total exposure as especially animal allergen levels can be much higher in schools compared to homes.

## Introduction

Indoor air quality influences respiratory health, especially in young children, as their lungs are still developing. Exposure to allergens, possibly in combination with other indoor agents like endotoxin and β-(1,3)-glucans, may lead to the development of allergy and allergic asthma and increased exposure to allergens worsens symptoms in children with allergy and allergic asthma. In studies investigating the effect of indoor exposure on health, the focus is often on the home environment. However, exposures in other environments where children frequently spend time may be just as important.

Besides the home environment, children spend a large part of their time in school buildings. Indoor air quality in schools may influence respiratory health, especially if the air quality differs from the home environment. For allergen exposure, some studies suggest that the home environment is a higher contributor of children’s’ exposure than schools [1]–[3], while other studies show higher exposure in schools compared to homes [1], [4], [5]. Endotoxin was only recently measured in schools and was higher in schools compared to homes [6], [7] while β-(1,3)-glucan measurements were only performed in two schools and was never compared to the home environment [8]. The conflicting relations between home and school exposure for allergen measurements may be influenced by climate or culture but can also be due to differences in measuring techniques. All these studies used settled vacuum floor dust [1]–[5], which may not be comparable between homes and schools, as school dust may contain more heavy particles like sand. The most representative matrix to measure for estimating respiratory exposure would be airborne dust but the necessary active measurements are expensive and invasive. Recently Noss et al [9] developed a low-cost devise to measure in settled airborne dust. This Electrostatic Dust fall Collector (EDC) captures settled dust at a height of at least 1.50 m, which may better represent inhalable air than vacuumed floor dust.

In the present study we evaluated school exposure in settled airborne dust to pet allergens, mouse allergens, house dust mite (HDM) allergens and β-(1,3)-glucans. We studied whether measured levels could be reproduced and explored factors related to the measured exposure levels. In addition we measured allergen and β-(1,3)-glucan in settled airborne dust in homes to compare the home and school environment. This was done in Dutch schools participating in the European project *Health Effects of Indoor Pollutants: Integrating microbial, toxicological and epidemiological approaches* (HITEA) and in homes of children attending these schools.

## Methods

### Selection of Schools and Homes

This study is a Dutch extension of the HITEA-project [10]. Selection of the schools is based on moisture and/or damp problems in the schools and is described in detail elsewhere [6], [10]. In short, for 79 Dutch schools (92 school buildings) a questionnaire on building characteristics and moisture and mould observations was filled in by school personnel (response rate: 35%). From those, 23 schools (27 school buildings) were selected for building inspections. For this Dutch extension of the HITEA study, exposure assessment was performed in 16 inspected schools that were willing to participate in this study. These 16 schools had in total 19 school buildings. After visual inspection of the schools [10], 8 buildings were characterised as buildings with mould or moisture problems and 11 buildings without.

**Figure 1:**
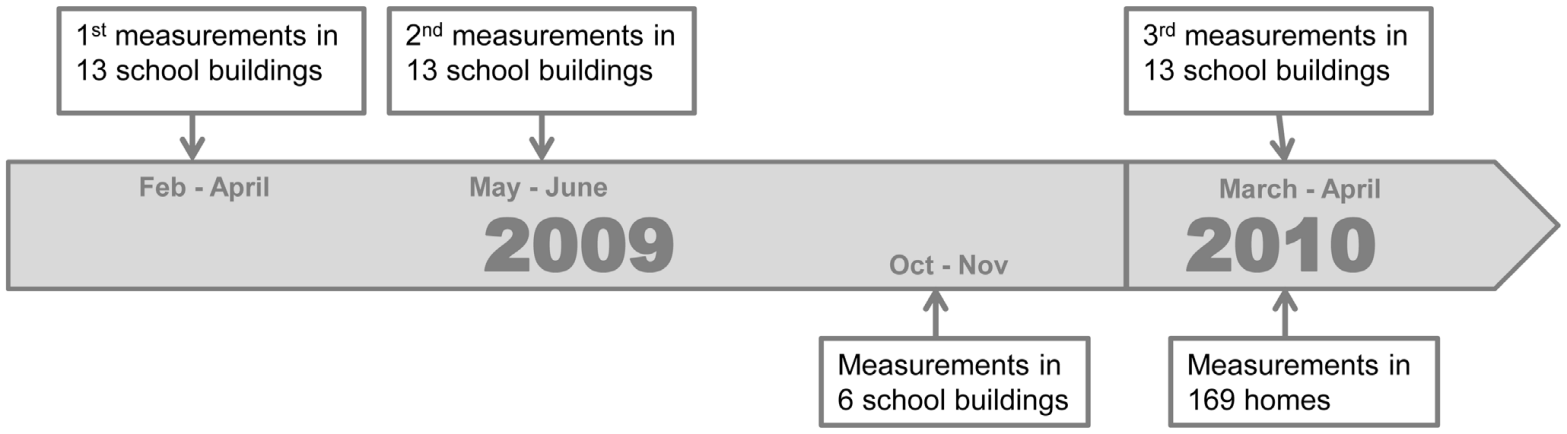
Study design. Measurements in schools were performed on 4 different occasions: winter 2009, spring 2009, fall 2009 and winter 2010. Exposure was measured in a total of 215 locations in 19 school buildings. Home measurements were performed all in winter 2010.

Ten schools (13 buildings) were selected for longitudinal exposure assessment in the HITEA study [6] and exposure was assessed 3 times in these schools. In the 6 other schools this was performed once (figure 1).

For the home measurements, 254 children attending the 10 schools in the longitudinal study were selected. They included 84 children with asthma-like symptoms and 170 children without, selected in a case-control design [6]. Children were considered cases if they reported one or more of the following: (1) wheezing/whistling in the past 12 months, (2) ever asthma, (3) medication use for asthma or wheezing or (4) dry cough at night apart from cold/infection in the past 12 months.

### Exposure Assessment

The EDC was used to collect settled airborne dust in both the schools and the homes [9], [11]. In schools the EDC was placed by study personnel at a height of at least 1.5 m, away from open windows, ventilation and heaters. They were placed at several locations in the schools including hallways, toilets, teachers lounges and part-time or full time used classrooms and collected dust for 8 weeks according to the HITEA exposure assessment protocol [6]. For the 13 school buildings from the longitudinal study, the collection was performed 3 times: in February-April 2009, in May/June 2009 and in March/April 2010 (figure 1). Each time, the EDC was placed at approximately the same spot. In the 6 additional schools, exposure was assessed in October/November 2009. Detailed questionnaires on building characteristics of the schools were available [10]. Percentage of pet owning children per class were calculated from HITEA health questionnaires filled in by the parents of the children [6], [12]. These percentages were available for 59% of the classrooms and from approximately 43% of the children in these classrooms. Socioeconomic status for a school was calculated as the mean years of education of the parents of the children that attended the school and filled in the questionnaire.

Home sampling took place in March/April 2010 (figure 1). The parents of the children were asked to fill in a short questionnaire on home characteristics. Parents received detailed instructions to place the EDC in the bedroom of the child at least 1.5 meter above the floor, away from windows, doors, heating and ventilation ducts. The sampler collected dust for two weeks according to a previously used home protocol [12]. After sampling, the parents carefully closed the EDC and returned it together with the questionnaire to our institute by mail.

The EDC cloth was sequentially extracted for allergens and β-(1,3)-glucans according to previously described protocols [12], [13]. Extract aliquots were stored at −20°C until analysis.

### Allergen and β-(1,3)-glucan Analysis

Cat, dog and mouse allergen levels were determined with the MARIA-assay (Indoor Biotechnologies Inc, Charlottesville, VA, USA) [14], [15] according to the manufacturers’ protocol. Concentration calculations by extrapolation of the standard curve done by the Bioplex software (Bio-Rad Laboratories, Hercules, CA, USA) down to 0.01 ng/ml for Fel d 1 and Can f 1, and to 1.5 pg/ml for Mus m 1 were accepted. Respectively 0.4%, 5.3% and 7.0% of the school samples and 54.2%, 75.0% and 79.8% of the home samples were below the limit of detection (LOD) for Fel d 1, Can f 1 and Mus m 1.

The MARIA assay for Der p 1 and Der f 1 (both house dust mite allergens) was not sensitive enough to determine these allergen levels in EDC extracts. To estimate mite levels, concentrations of domestic mite (DM) antigens were determined with a new sensitive enzyme immunoassay based on polyclonal rabbit antibodies to *Dermatophagoides farinae* protein [16]. The LOD of this assay was 0.05 ng/ml. No school samples and five home samples (3%) had levels below LOD.

Βeta-(1,3)-glucan levels in the extracts were determined with the β-(1,3)-glucan sandwich assay described by Noss et al [13]. The LOD was 0.2 ng/ml and only one sample (from a home) had a level below LOD.

Values below LOD were assigned a value of 2/3 of the LOD. All values were back calculated to ng/m^2^ and to express all results as ng/m^2^/week, home values were divided by 2 weeks and schools by 8 weeks.

### Ethics Statement

The protocol of the Dutch part of the HITEA study was approved by the medical ethical testing commission from the Utrecht University Medical Centre. Parents and children over 11 years of age gave written informed consent.

### Statistical Analysis

All analyses were performed using SAS (version 9.2). Exposure levels were log-transformed. Results from the repeated measurements were analysed using generalised linear models (GLM).

The influence of variables on the measured levels in schools was determined for classrooms. For all schools, the latest measurements were included (Fall 2009 or Winter 2010). For this, the MIXED procedure, a generalization of standard linear models, was used. This procedure allows 2-level models and all models, including those for home exposure, were adjusted for ‘school’ to correct for possible clustering. All parameters with an univariate p value less than 0.20 were considered in multivariate testing.

The comparison between the home and school environment was performed with Students’ T-test. P-values below 0.05 were considered statistically significant.

## Results

### Exposure in Schools

Levels of domestic mite (DM) antigen, cat allergen (Fel d 1), dog allergen (Can f 1), mouse allergen (Mus m1) and β-(1,3)-glucans were measured for a total of 227 different locations in 19 school buildings, including hallways, toilets, teachers lounges and part-time or full-time used classrooms. Characteristics of the school buildings are in table 1. We found the highest levels for all allergens and glucans in full-time used classrooms and hallways. The levels in these classrooms and hallways were comparable and about two times higher than in teachers rooms, bathrooms and part-time used classrooms for all analytes.

**Table 1:** Characteristics and measured exposure of the schools (latest measurement used).

	All school buildings:	Buildings measuredin fall 2009	Buildings measuredin winter 2010
Number	19	6	13
Total number of classrooms	123	88	35
Moisture damaged school building	8	3	5
Age of the building (years, range)	30 (2–79)	36 (2–79)	28 (12–69)
Number of floor levels (range)	1–3	1–2	1–3
Socioeconomic status (mean years education of parents, SD)	14.4 (2.6)	15.0 (1.5)	13.6 (3.9)
Self-reported pet ownership per class (mean %)	38%	30%	43%
Self-reported cat ownership per class (mean %)	25%	16%	21%
Self-reported dog ownership per class (mean %)	19%	22%	15%
Class room levels of:			
β (1,3) glucans (ng/m^2^/week, GM (GSD))	1258 (1.88)	1345 (2.33)	1211 (1.72)
DM antigen (ng/m^2^/week, GM (GSD))	133.5 (2.59)	257.5 (2.99)	104.6 (2.23)
Cat allergen (ng/m^2^/week, GM (GSD))	60.6 (2.45)	38.6 (3.46)	74.3 (1.90)
Dog allergen (ng/m^2^/week, GM (GSD))	35.8 (3.34)	14.3 (5.81)	53.8 (1.85)
Mouse allergen (ng/m^2^/week, GM (GSD))	1.16 (3.04)	1.0 (2.13)	1.3 (3.33)

SD: standard deviation.

GM: geometric mean.

GSD: geometric standard deviation.

In 13 school buildings from 10 schools, measurements were performed three times (figure 1). Repeated measurement of different types of locations within the same year correlated moderately well, with levels of DM and Can f 1 significantly decreasing in the spring compared to winter (table 2). Although the measurements that were performed one year later still correlated with the previous measurements, mean levels were significantly different for all analytes (table 2).

**Table 2:** Repeated measurements in 13 school buildings (all location types).

		Winter 2009		Spring 2009		Winter 2010		Correlationbetween winterand spring 2009		Correlationbetween winter2009 and 2010	
	N	Geom.mean	Geom.SD	Geom.mean	Geom.SD	Geom.mean	Geom.SD				
		(ng/m^2^/week)		(ng/m^2^/week)		(ng/m^2^/week)		r	p	r	p
HDM	117	177.7	2.249	140.8*	2.588	92.1*	2.404	0.503	<0.001	0.508	<0.001
β-glucans	126	1313	1.950	1281	1.923	1075*	1.862	0.568	<0.001	0.607	<0.001
Fel d 1	99	49.7	1.866	50.2	2.080	67.8*	1.950	0.637	<0.001	0.268	0.007
Can f 1	89	31.8	2.004	17.4*	2.046	48.9*	1.945	0.586	<0.001	0.450	<0.001
Mus m 1	130	Not done		0.76	4.699	1.20*	3.784	0.686†	<0.001		

* Significantly different (p≤0.002) from winter 2009 or (for Mus m 1) from previous measurement.

† Correlation between the 2 measurements (spring 2009 and winter 2010).

For analyses of determinants of exposure only results from the full-time used classrooms were considered. From the 10 schools that were measured repeatedly (figure 1), only the third time point was included for analysis of all schools. Measurements and characteristics were available for 123 full-time used classrooms from a total of 19 school buildings of 16 schools. Mean levels of the different analytes in classrooms are in table 1. For Fel d 1 the within school variance was higher than the between school variance while for all other analytes the between school variance is higher than the within school variance.

The influences of moisture status of the school, measuring period, observed moisture problems in the classroom, age of the building, floor level, grade, occupancy of the classroom and percentage of children with a specific pet on the measured levels were studied. As on average pet owning data from the health questionnaire was available from 12 children per class (43%), actual numbers of pet owning children per class were unknown. From 41% of the classrooms pet ownership was completely unknown. Results from univariate analysis are in table 3. None of the studied variables significantly influenced the Mus m 1 levels in classrooms but if only measurement performed in the winter were considered, the moisture status of the building influenced Mus m 1 levels (Geometric Mean ratio: 1.68, 95% Confidence Interval: 1.15–2.45).

**Table 3:** Univariate and multivariate analyses of classroom measurements.

	β (1,3) glucans	DM antigen	Cat allergen	Dog allergen	Mouse allergen
	GMR (95% CI)	GMR (95% CI)	GMR (95% CI)	GMR (95% CI)	GMR (95% CI)
Full time used classrooms (compared to other locations)	**1.23 (1.15–1.32)**	**1.15 (1.04–1.27)**	**1.19 (1.09–1.30)**	**1.25 (1.16–1.36)**	**1.13 (1.02–1.26)**
*Univariate analysis on classrooms*					
Season (sampled in winter (ref) vs. fall)	1.02 (0.83–1.26)	**1.44 (1.10–1.88)**	**0.74 (0.57–0.97)**	**0.53 (0.35–0.80)**	0.90 (0.63–1.31)
Moisture status school	**1.24 (1.05–1.47)**	*1.26 (0.96–1.66)*	1.15 (0.88–1.50)	*1.37 (0.87–2.16)*	*1.25 (0.90–1.74)*
Moisture/mold observations in classroom	0.93 (0.78–1.09)	*1.27 (0.98–1.65)*	0.94 (0.74–1.19)	0.96 (0.78–1.18)	*0.81 (0.63–1.05)*
Age of building (per 10 years increase)	*1.03 (0.99–1.07)*	*1.05 (0.99–1.12)*	1.00 (0.93–1.06)	1.00 (0.89–1.12)	1.02 (0.94–1.10)
Floor level	**0.90 (0.82–0.98)**	**0.80 (0.70–0.91)**	0.96 (0.85–1.09)	1.00 (0.88–1.13)	0.97 (0.84–1.12)
Occupancy (per 5 students increase)	**1.06 (1.00–1.12)**	1.04 (0.95–1.15)	*1.07 (0.98*–*1.16)*	*1.08 (1.01*–*1.16)*	*1.10 (0.98*–*1.24)*
Pet owning children (per 5% increase)	1.00 (0.99–1.02)	1.00 (0.98–1.02)	**1.03 (1.01–1.05)**	1.01 (0.99–1.03)	*1.02 (0.99*–*1.04)*
Cat owning children (per 5% increase)	1.00 (0.99–1.02)	1.00 (0.97–1.03)	*1.03 (1.00*–*1.05)*	0.99 (0.96–1.02)	1.01 (0.98–1.05)
Dog owning children (per 5% increase)	1.00 (0.98–1.02)	0.99 (0.96–1.02)	*1.03 (1.00*–*1.06)*	**1.05 (1.02–1.08)**	1.02 (0.98–1.05)
Higher grade (1 grade increase)	*0.98 (0.96*–*1.00)*	**0.54 (0.53–0.56)**	*0.99 (0.96*–*1.02)*	1.00 (0.98–1.03)	*0.97 (0.94*–*1.01)*
Socioecononic status	**1.06 (1.03–1.09)**	**1.06 (1.01–1.12)**	**1.10 (1.06–1.14)**	**1.15 (1.06–1.24)**	*0.95 (0.89*–*1.02)*
*Multivariate analysis on classrooms*					
Season (sampled in winter (ref) vs. fall)		1.61 (1.35–1.92)	NS	NS	
Moisture status school	1.67 (1.02–1.33)	NS		NS	NS
Moisture/mold observations in classroom		NS			NS
Age of building (per 10 years increase)	NS	NS			
Floor level	0.91 (0.84–0.99)	0.86 (0.76–0.97)			
Occupancy (per 5 students increase)	NS		NS	NS	NS
Pet owning children (per 5% increase)			NS		NS
Cat owning children (per 5% increase)			1.03 (>1.00–1.06)		
Dog owning children (per 5% increase)			1.03 (1.01–1.06)	2.26 (1.22–4.20)	
Higher grade (1 grade increase)		0.96 (0.93–0.99)	NS		NS
Socioecononic status	1.06 (1.03–1.08)	1.08 (1.04–1.11)	1.09 (1.05–1.14)	1.15 (1.08–1.22)	NS

GMR: Geometric Mean Ratio.

95%CI: 95% Confidence Interval.

In **bold** are significant univariate associations that are considered in multivariate testing.

*Italic*: univariate analysis with p<0.20 and considered in multivariate testing.

NS: variables were included in multivariate testing and found to be not significant.

Empty: the variable was not included in the specific multivariate model.

Results of multivariate analyses are also in table 3. We found β-(1,3)-glucan levels to be significantly higher in schools with moisture problems and with higher socioeconomic status while lower in rooms on higher floors. For DM, levels were significantly higher in the buildings measured in fall compared to winter and schools with higher socioeconomic status while grade and floor level had a significant negative effect. Fel d 1 levels were significantly influenced by socioeconomic status and the percentage of cat and dog owners. The percentage of dog owning children was related to Can f 1 levels and these levels were also influenced by socioeconomic status. None of the studied variables significantly influenced the Mus m 1 levels in classrooms.

### Exposure in Homes

A total of 169 EDC’s (67%) were returned and analysed, while home characteristics, were available from 168 homes. Home characteristics are in table 4. Overall, 48% of the children had a pet in the house, including rabbits and some exotic rodents; 18% had a dog, 23% a cat and none of the children kept mice as pets. Data from a questionnaire from a previous part of HITEA was used to compare responders and non-responders. For most household characteristics, responders and non-responders did not differ but parents of non-responders smoked more often in the home. In addition, they also reported more mould odour although no significant difference was found in the reporting of mould or moisture observations.

**Table 4:** Home characteristics and exposure levels.

	Homes
EDC measurements in homes (N)	169
Home questionnaires available (N)	168
Homes of children with asthmatic symptoms (N)	65 (38%)
Age of the building (years, mean, range)	32 (3–160)
Homes with self-reported moisture/mould observations (N)	48 (28%)
Occupancy (mean, range)	4.2 (2–7)
Reported smoking in the home (N, %)	18 (11%)
Socioeconomic status (mean years of parentaleducation, range)	15,8 (6.5–24)
Reported having pets (N, %)	81 (48%)
Reported having a cat (N, %)	39 (23%)
Reported having a dog (N, %)	29 (17%)
Types of floors	
Smooth floor (N, %)	80 (48%)
Smooth floor with carpet (N, %)	49 (29%)
Carpet (N, %)	38 (23%)
Exposure to β-(1,3)-glucans (ng/m2/week, GM, GSD)	
β-(1,3)-glucans (ng/m2/week, GM, GSD)	684.3 (2.265)
HDM (ng/m2/week, GM, GSD)	333.9 (4.018)
Fel d 1 (all homes, ng/m2/week, GM, GSD)	13.3 (6.516)
Fel d 1 (homes without cat, ng/m2/week, GM, GSD)	5.6 (2.535)
Fel d 1 (homes with cat, ng/m2/week, GM, GSD)	235.7 (3.373)*
Can f 1 (all homes, ng/m2/week, GM, GSD)	7.4 (5.012)
Can f 1 (homes without dog, ng/m2/week, GM, GSD)	3.9 (1.954)
Can f 1 (homes with dog, ng/m2/week, GM, GSD)	163.5 (3.069)*
Mus m 1 (all homes, ng/m2/week, GM, GSD)	0.63 (1.954)
Mus m 1 (homes without pet, ng/m2/week, GM, GSD)	0.54 (1.552)
Mus m 1 (homes with pet, ng/m2/week, GM, GSD)	0.73 (2.280)†

† significantly different from homes without pets, p = 0.004.

* significantly different from homes without the specific pet, p<0.001.

Geom. SD: geometric standard deviation.

Bedroom samples were collected in a case-control design and therefore differences between levels in bedrooms of children with and without asthma-like symptoms were explored. No significant differences were found for measured levels of cat allergen, DM antigen, mouse allergen and β-(1,3)-glucans (table 5). Exposure to dog allergen tended to be higher in the bedrooms of children with asthma-like symptoms compared to bedrooms of children without asthma-like symptoms (10.1 vs. 6.1 ng/m^2^/week, p = 0.069) even though no differences were found in the frequency of pet ownership between these children. There were also no differences in the frequency that the pets were allowed in the bedrooms or self-reported cleaning frequency between the two groups.

**Table 5:** Univariate and multivariate analyses of bedroom samples.

	β (1,3) glucans	DM antigen	Cat allergen	Dog allergen	Mouse allergen
	GMR (95% CI)	GMR (95% CI)	GMR (95% CI)	GMR (95% CI)	GMR (95% CI)
*Univariate analysis on bedrooms*					
Childs asthma status	1.03 (0.93–1.15)	1.11 (0.93–1.33)	0.87 (0.68–1.12)	*1.23 (0.99*–*1.52)*	1.04 (0.95–1.14)
Moisture/mold observations	1.04 (0.93–1.17)	1.01 (0.83–1.23)	0.92 (0.70–1.21)	1.12 (0.86–1.41)	0.96 (0.87–1.06)
Higher grade at school	**1.04 (>1.00–1.08)**	*1.05 (0.99*–*1.12)*	0.96 (0.88–1.05)	1.00 (0.93–1.08)	0.99 (0.95–1.02)
Occupancy (1 person increase)	**1.07 (>1.00–1.15)**	**1.21 (1.08–1.35)**	1.03 (0.87–1.21)	1.03 (0.90–1.19)	1.02 (0.96–1.08)
Age of building (per 10 years increase)	1.00 (0.97–1.02)	1.02 (0.98–1.07)	**1.07 (1.01–1.12)**	1.00 (0.95–1.05)	1.01 (0.99–1.03)
Smoking in the house	1.00 (0.83–1.19)	*0.82 (0.60–1.10)*	1.07 (0.71–1.62)	**1.44 (1.02–2.05)**	1.04 (0.90–1.20)
Pets in the home	0.94 (0.85–1.05)	1.02 (0.85–1.22)	**2.15 (1.73–2.67)**	**1.79 (1.48–2.17)**	**1.17 (1.05–1.24)**
Cats in the home	0.94 (0.83–1.06)	1.08 (0.88–1.33)	**5.06 (4.35–5.89)**	1.00 (0.78–1.28)	*1.11 (1.00–1.23)*
Dogs in the home	0.97 (0.84–1.12)	*0.83 (0.66–1.06)*	1.02 (0.73–1.41)	**5.09 (4.45–5.81)**	1.06 (0.94–1.19)
Socioeconomic status(mean years of education of the parents)	**1.02 (>1.00–1.04)**	1.01 (0.98–1.05)	1.02 (0.98–1.07)	0.98 (0.94–1.02)	1.00 (0.98–1.01)
Type of floor:					
Smooth floor without carpet	ref.	ref.	ref.	ref.	ref.
Smooth floor with carpet	1.06 (0.93–1.20)	1.24 (1.00–1.52)	1.00 (0.75–1.33)	0.81 (0.64–1.04)	0.97 (0.88–1.08)
Carpet	0.94 (0.82–1.07)	1.08 (0.86–1.36)	1.08 (0.79–1.48)	*0.99 (0.76–1.30)*	1.03 (0.92–1.15)
*Multivariate analysis on bedrooms*					
Childs asthma status				NS	
Higher grade at school	NS	NS			
Occupancy (1 person increase)	NS	1.21 (1.08–1.35)			
Age of building (per 10 years increase)			NS		
Smoking in the house		NS		NS	
Pets in the home			NS	NS	1.17 (1.05–1.24)
Cats in the home			5.06 (4.35–5.89)		NS
Dogs in the home		NS		5.09 (4.45–5.81)	
Socioeconomic status(mean years of education of the parents)	1.02 (>1.00–1.04)				
Type of floor				NS	

GMR: Geometric Mean Ratio.

95%CI: 95% Confidence Interval.

In **bold** are significant univariate associations that are considered in multivariate testing.

*Italic*: univariate analysis with p<0.20 and considered in multivariate testing.

NS: variables were included in multivariate testing and found to be not significant.

Empty: the variable was not included in the specific multivariate model.

Average levels measured in the bedrooms are in table 4. As expected, cat allergen levels were significantly higher in homes with a cat while dog allergen levels were significantly higher in homes with a dog. Associations between household characteristics and exposure are in table 5. Although nobody reported to keep mice as pets, significantly higher levels of Mus m 1 were found in bedrooms of pet owners (table 4). Results of multivariate analyses are shown in table 5. Fel d 1 levels were only associated with cat ownership, Can f 1 levels only with dog ownership and Mus m 1 levels with pet ownership. For β-(1,3)-glucans only the educational level of the parents had a significant influence while for DM antigen only occupancy was significant.

### Schools vs. Homes

**Figure 2:**
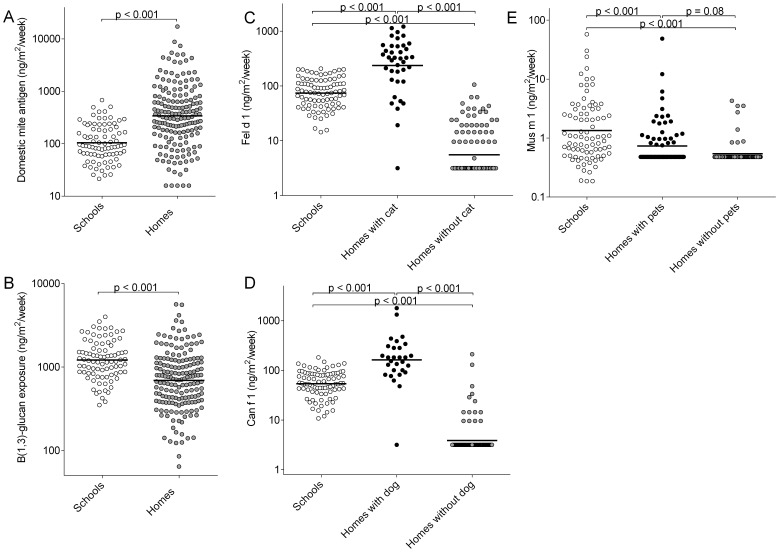
β-(1,3)-glucan and allergen levels in schools and homes in winter 2010. Measured levels of domestic mite antigen (A), β-(1,3)-glucans (B), cat allergen (C), dog allergen (D) and mouse allergen (E) in classrooms (n = 87, open circles) and bedrooms (n = 169, filled circles). Homes with and without pets are separately shown for the animal derived allergens.

As the measured levels in classrooms were influenced by the measurement period, only school measurements conducted in the winter of 2010 (13 school buildings, 87 classrooms) were compared to the home measurements performed in winter 2010 (figure 1). Figure 2 shows the differences in levels between homes and schools. Only the DM level was highest in bedroom samples while for cat, dog and mouse allergens and β-(1,3)-glucans classroom samples were up to 13 times higher compared to bedrooms.

## Discussion

In this study we showed variation of exposure to allergens and β-(1,3)-glucans over time in schools. Exposure in schools was explained by pet ownership of the children attending the schools, the socioeconomic status of these children, floor level of the classroom and grade. Socioeconomic status and pet ownership also influenced the exposures measured in the homes while in homes also occupancy contributed. Animal allergen and β-(1,3)-glucan levels in settled airborne dust were overall higher in classrooms compared to children’s bedroom while DM antigens were found in higher levels in the bedrooms.

The house dust mite allergens Der p 1 and Der f 1 could not be measured with the MARIA in EDC extracts as over 98% of the samples in our study had levels below the limit of detection. Therefore the more sensitive domestic mite antigen assay was used as a marker for house dust mite levels [16]. It was shown that the DM antigen quantities correlated well with the sum of Der p 1 and Der f 1 values but we realize we measure DM antigen and not the actual allergen. Although unlikely, it is possible that the composition of the DM fraction measured in homes is different from that measured in schools with regard to the allergen fraction within the antigens, which could result in additional differences in exposure between homes and schools. Also, house dust mite allergens are often found in relatively larger particles that may not easily become airborne and may not reach the EDC. Our results may therefore not represent the overall availability of the dust mite antigens but more the inhaled fraction.

There was a difference in sampling strategy between the homes and the schools. Schools were sampled for 8 weeks, according to the HITEA protocol, while homes were samples for 2 weeks, according to a previously used home protocol [13]. The extended sampling period in schools was used to synchronize the dust collection with other exposure measurements and to reduce the inconvenience for the schools. We adjusted all our results for sampling time but a possible influence of the two sampling procedures on the results cannot be excluded. Previously it was shown that increased exposure time resulted in a less than linear increase of concentrations for endotoxin while for β-(1,3)-glucans the increase in exposure was more linear [13]. Extraction inefficiency with higher dust levels or resuspension of the dust on the EDC to the air, resulting in a steady state without dust increase over time, may have caused this possible non-linear relationship [13]. If in our study the same reduced efficiency with prolonged exposure would have occurred, it would have resulted in an underestimation of the school levels, making the real differences for animal allergens and glucans between home and school levels even bigger than found.

We assessed a large school sample, with 215 measured locations in 19 school buildings. However, the study design, with index and reference schools, may have resulted in an overrepresentation of index schools. Index status of the schools had a positive effect on the exposure to β-(1,3)-glucans, components of fungi that are related to moisture and mold problems. For β-(1,3)-glucans, there may be an effect of overrepresentation of the index schools while for the other exposures we found no significant effect of school index status, indicating that the effect of this overrepresentation may be minor. In the homes, we measured only in bedrooms of children. This is probably the place where the children spend relatively the most time, followed by the living room. Previous studies have shown for measurements in floor dust that allergen levels of pet allergens [17] and house dust mite allergens [18] in different areas of the house are comparable but such a relation was not found for β-(1,3)-glucans [19].

Like some other studies [2], [4], [5], we found pet allergen levels to be higher in classrooms than in most homes. The significant indicator for cat and dog allergens in classrooms and homes in our study was the presence of children who had pets at their home. For classroom exposure, pet ownership was based on the questionnaires filled in by the parents. This questionnaire was distributed under children age 6 years and older [12] while children from the age 4 start attending school in The Netherlands. Therefore we missed data of 41% of the assessed classrooms. Missing data in the questionnaire on classroom level resulted in pet ownership based on 43% of the children in the room only, which may influence our results. However, previous studies also showed relationships between pet ownership and pet allergens in classrooms [20], [21]. Cat and dog allergen levels in classrooms were found to be high enough to induce allergic symptoms in children [21], [22]. The route of transport of the allergens is probably the clothing of the pet owning children [23] and also the hair may be a route of allergen transport [24], [25]. Via these transport ways other children may bring the allergens from school to homes without pets, thus increasing home levels [25].

Repeated exposure assessment in the schools showed that locations with high exposure remained high over time. However, the actual levels differed significantly over time. In contrast, exposure to endotoxin in these schools was stable over the 3 measuring periods [26]. Differences between measurements in winter and spring may be explained by ventilation differences in these seasons as well as by temperature and humidity differences. We also found a difference between the measurements performed in succeeding winters. This may be explained by the composition of the children in the classroom which changes every September.

The EDC is a passive collector for settled airborne dust which has not been used in classrooms before. It is therefore difficult to compare the results to previous school studies performed on reservoir dust, collected with vacuum cleaners. Results from petri dish methods may be comparable to the EDC as they also collect settled airborne dust but only few school samples had been determined and the results were expressed per gram dust [27]–[29], making it also impossible to compare to our EDC results. A previous study in a companion animal hospital using the EDC found cat allergen levels comparable with the levels found in classrooms [11]. Dog allergens in classrooms were lower than found in this clinic but levels are still comparable to levels found in the animal operating room.

In multivariate analyses for schools we found that moisture status was related to β-(1,3)-glucans exposure. Moisture status of the school is probably related to mold growth, resulting in glucan exposure. We found reduced house dust mite antigen in winter compared to fall, probably due to decreased temperatures and humidity in winter. Socioeconomic status, in the HITEA study defined as the mean years of education of the parent of the children, was positively related to almost all exposures, even after adjustment for other factors. Commonly in The Netherlands, the municipality is responsible for maintenance of school buildings, without financial contribution of the parents. In homes it has been shown before that socioeconomic status influences allergen exposure [30], [31]. These studies showed increased exposure to HDM and pet allergens in homes with higher socioeconomic status. An increased transfer of allergens and β-(1,3)-glucans from homes with higher socioeconomic status to the schools may explain our findings. However, in the homes we assessed we only found a relation between β-(1,3)-glucans and socioeconomic status. HDM exposure at home was related to the amount of people living in the home which is not surprising as these mite thrive in beds and more family members means more beds. This is however in contrast to findings of Leaderer et al [31] who showed elevated HDM levels in homes with less people. Animal allergen exposure in the homes was related to animals present. Surprisingly, mouse allergen levels were higher in homes with pets. Possible the availability of animal food in the home increases the risk of mouse infestation.

Previous comparisons of allergen levels between homes and schools [1]–[5] have all been performed on samples from vacuumed floor dust. Floor dust from schools may contain more sand from the playground, which makes the dust heavier. This makes comparison of levels expressed per gram between homes and schools in vacuumed dust difficult as home dust may contain less heavy soil material. We measured in settled airborne dust, which is not influenced by soil material brought in from outside and therefore may be better suitable for a comparison between different environments. Two recent studies in the US also compared animal allergen levels in homes to levels in schools [1], [5] and also found school levels to be higher. However, levels of mouse allergen found in these studies were significantly higher than the cat or dog allergen levels, while in our study the amount of mouse allergens in both environments was very low. Muti et al [32] showed also low mouse allergen levels in Italy, indicating that the situation in Europe and the US may not be comparable.

This study measured for the first time β-(1,3)-glucan exposure in a large school sample. Βeta-(1,3)-glucans, possibly in combination with other microbial structures, may reduce the risk for atopy and asthma [33]. We previously reported that endotoxin levels in schools were related to respiratory symptoms [6], while endotoxin levels in other environments may be protective [34]. The role of relatively high levels of β-(1,3)-glucans on respiratory health in children remains to be determined. In comparison to one previous study on β-(1,3)-glucan levels in settled airborne dust in homes of students [35], our homes had much lower levels possibly because the type of housing and cleaning frequency was very different. Studies on vacuumed floor dust from homes [36]–[38] show much higher levels per m^2^ compared to our passive sampler. Although EDC levels were shown to relate to floor dust levels, EDC levels are lower because they contain less heavy and large particles [35]. Comparisons between homes and schools have not been done before. The only known measurements in schools were performed in floor dust from two schools [8] and levels up to 100 times higher compared to ours were found. We believe that results from our EDC’s may be more representative for the inhaled air in schools than these previous results.

Major birth cohort studies focusing on exposure-response relations often use home exposure as determinant for total exposure [33], [34], [36]. The present study shows that children at school are exposed to about 2 times higher levels of β-(1,3)-glucans and mouse allergens compared to the home environment and up to 13 times higher levels of pet allergens. Compared to the school environment, animal allergen levels in homes with a specific pet are only about 3 times higher. Dutch children are at school about 30 hours per week while they spend 4 to 5 times more time in their home. This means that indoor exposure in schools to especially pet allergens, but also to mouse allergens and glucans, substantially contributes to children’s total exposure. For mite exposure, the home environment could be considered as the major contributor.

In summary, we showed that measurements of school exposure in different seasons or years can give different results for both the allergens and β-(1,3)-glucans. Mayor contributors for school exposure are moisture status of the school as well as socioeconomic status and pet ownership of the attending children. In addition, our results show high levels of cat, dog and mouse allergens as well as β-(1,3)-glucans in settled airborne dust in classrooms compared to bedrooms. β-(1,3)-glucan and pet allergen levels in schools could contribute substantially to the total exposure of children, especially for children without pets at home.

## References

[1] Sheehan WJ, Rangsithienchai PA, Muilenberg ML, Rogers CA, Lane JP, et al (2009) Mouse allergens in urban elementary schools and homes of children with asthma. Ann Allergy Asthma Immunol 102: 125–130. 19230463 10.1016/S1081-1206(10)60242-6 PMC2658645

[2] Dotterud LK, Van TD, Kvammen B, Dybendal T, Elsayed S, et al (1997) Allergen content in dust from homes and schools in northern Norway in relation to sensitization and allergy symptoms in schoolchildren. Clin Exp Allergy 27: 252–261. 9088651

[3] Zhang L, Chew FT, Soh SY, Yi FC, Law SY, et al (1997) Prevalence and distribution of indoor allergens in Singapore. Clin Exp Allergy 27: 876–885. 9291283

[4] Perzanowski MS, Ronmark E, Nold B, Lundback B, Platts-Mills TA (1999) Relevance of allergens from cats and dogs to asthma in the northernmost province of Sweden: Schools as a major site of exposure. J Allergy Clin Immunol 103: 1018–1024. 10359880 10.1016/s0091-6749(99)70173-9

[5] Permaul P, Hoffman E, Fu C, Sheehan W, Baxi S, et al (2012) Allergens in urban schools and homes of children with asthma. Pediatr Allergy Immunol 23: 543–549. 22672325 10.1111/j.1399-3038.2012.01327.x PMC3424376

[6] Jacobs JH, Krop EJ, Wind SD, Spithoven J, Heederik DJ (2013) Endotoxin levels in homes and classrooms of Dutch school children and respiratory health. Eur Respir J 42: 314–322. 23100494 10.1183/09031936.00084612

[7] Sheehan WJ, Hoffman EB, Fu C, Baxi SN, Bailey A, et al (2012) Endotoxin exposure in inner-city schools and homes of children with asthma. Ann Allergy Asthma Immunol 108: 418–422. 22626594 10.1016/j.anai.2012.04.003 PMC3367391

[8] Foarde K, Berry M (2004) Comparison of biocontaminant levels associated with hard vs. carpet floors in nonproblem schools: Results of a year long study. J Expo Anal Environ Epidemiol 14 Suppl 1S41–8. 15118744 10.1038/sj.jea.7500357

[9] Noss I, Wouters IM, Visser M, Heederik DJ, Thorne PS, et al (2008) Evaluation of a low-cost electrostatic dust fall collector for indoor air endotoxin exposure assessment. Appl Environ Microbiol 74: 5621–5627. 18676704 10.1128/AEM.00619-08 PMC2547045

[10] Haverinen-Shaughnessy U, Borras-Santos A, Turunen M, Zock JP, Jacobs J, et al (2012) Occurrence of moisture problems in schools in three countries from different climatic regions of Europe based on questionnaires and building inspections - the HITEA study. Indoor Air 22: 457–466. 22404345 10.1111/j.1600-0668.2012.00780.x

[11] Samadi S, Heederik DJ, Krop EJ, Jamshidifard AR, Willemse T, et al (2010) Allergen and endotoxin exposure in a companion animal hospital. Occup Environ Med 67: 486–492. 20519747 10.1136/oem.2009.051342

[12] Borràs-Santos A, Jacobs JH, Täubel M, Haverinen-Shaughnessy U, Krop EJ, et al (2013) Dampness and mould in schools and respiratory symptoms in children: the HITEA study. Occup Environ Med 70: 681–687. 23775866 10.1136/oemed-2012-101286

[13] Noss I, Doekes G, Sander I, Heederik DJ, Thorne PS, et al (2010) Passive airborne dust sampling with the electrostatic dustfall collector: Optimization of storage and extraction procedures for endotoxin and glucan measurement. Ann Occup Hyg 54: 651–658. 20354054 10.1093/annhyg/meq026

[14] Earle CD, King EM, Tsay A, Pittman K, Saric B, et al (2007) High-throughput fluorescent multiplex array for indoor allergen exposure assessment. J Allergy Clin Immunol. 119: 428–433. 10.1016/j.jaci.2006.11.004 17196246

[15] King EM, Filep S, Smith B, Platts-Mills T, Hamilton RG, et al (2013) A multi-center ring trial of allergen analysis using fluorescent multiplex array technology. J Immunol Methods. 387: 89–95. 10.1016/j.jim.2012.09.015 PMC3955085 23085532

[16] Sander I, Zahradnik E, Kraus G, Mayer S, Neumann HD, et al.. (2012) Domestic mite antigens in floor and airborne dust at workplaces in comparison to living areas: A new immunoassay to assess personal airborne allergen exposure. PLoS One 7: doi: 10.1371/journal.pone.0052981. 10.1371/journal.pone.0052981 PMC3528730 23285240

[17] Arbes SJ Jr, Cohn RD, Yin M, Muilenberg ML, Friedman W, et al (2004) Dog allergen (can f 1) and cat allergen (fel d 1) in US homes: Results from the national survey of lead and allergens in housing. J Allergy Clin Immunol 114: 111–117. 15241352 10.1016/j.jaci.2004.04.036

[18] Simpson A, Simpson B, Custovic A, Cain G, Craven M, et al (2002) Household characteristics and mite allergen levels in Manchester, UK. Clin Exp Allergy 32: 1413–1419. 12372118 10.1046/j.1365-2745.2002.01496.x

[19] Douwes J, Zuidhof A, Doekes G, van der Zee SC, Wouters I, et al (2000) (1–>3)-beta-D-glucan and endotoxin in house dust and peak flow variability in children. Am J Respir Crit Care Med 162: 1348–1354. 11029343 10.1164/ajrccm.162.4.9909118

[20] Almqvist C, Larsson PH, Egmar AC, Hedren M, Malmberg P, et al (1999) School as a risk environment for children allergic to cats and a site for transfer of cat allergen to homes. J Allergy Clin Immunol 103: 1012–1017. 10359879 10.1016/s0091-6749(99)70172-7

[21] Almqvist C, Wickman M, Perfetti L, Berglind N, Renstrom A, et al (2001) Worsening of asthma in children allergic to cats, after indirect exposure to cat at school. Am J Respir Crit Care Med 163: 694–698. 11254526 10.1164/ajrccm.163.3.2006114

[22] Munir AK, Einarsson R, Schou C, Dreborg SK (1993) Allergens in school dust. I. the amount of the major cat (fel d I) and dog (can f I) allergens in dust from Swedish schools is high enough to probably cause perennial symptoms in most children with asthma who are sensitized to cat and dog. J Allergy Clin Immunol 91: 1067–1074. 8491939 10.1016/0091-6749(93)90221-z

[23] Berge M, Munir AK, Dreborg S (1998) Concentrations of cat (fel d1), dog (can f1) and mite (der f1 and der p1) allergens in the clothing and school environment of Swedish schoolchildren with and without pets at home. Pediatr Allergy Immunol 9: 25–30. 9560839 10.1111/j.1399-3038.1998.tb00296.x

[24] Krop EJ, Doekes G, Stone MJ, Aalberse RC, van der Zee JS (2007) Spreading of occupational allergens: Laboratory animal allergens on hair-covering caps and in mattress dust of laboratory animal workers. Occup Environ Med 64: 267–272. 17053016 10.1136/oem.2006.028845 PMC2078456

[25] Karlsson AS, Renstrom A (2005) Human hair is a potential source of cat allergen contamination of ambient air. Allergy 60: 961–964. 15932389 10.1111/j.1398-9995.2005.00796.x

[26] Jacobs JH, Krop EJ, Borras-Santos A, Zock JP, Taubel M, et al.. (2003) Endotoxin levels in settled airborne dust in European schools: the HITEA school study. Indoor Air: doi: 10.1111/ina.12064. 10.1111/ina.12064 23927557

[27] Karlsson AS, Renström A, Hedrén M, Larsson K (2002) Comparison of four allergen-sampling methods in conventional and allergy prevention classrooms. Clin Exp Allergy 32: 1776–1781. 12653171 10.1046/j.1365-2222.2002.01553.x

[28] Karlsson AS, Hedrén M, Almqvist C, Larsson K, Renström A (2002) Evaluation of Petri dish sampling for assessment of cat allergen in airborne dust. Allergy 57: 164–168. 11929422 10.1034/j.1398-9995.2002.1s3297.x

[29] Zhao ZH, Elfman L, Wang ZH, Zhang Z, Norbäck D (2006) A comparative study of asthma, pollen, cat and dog allergy among pupils and allergen levels in schools in Taiyuan city, China, and Uppsala, Sweden. Indoor Air 16: 404–413. 17100662 10.1111/j.1600-0668.2006.00433.x

[30] Kitch BT, Chew G, Burge HA, Muilenberg ML, Weiss ST, et al (2000) Socioeconomic predictors of high allergen levels in homes in the greater Boston area. Environ Health Perspect 108: 301–307. 10753087 10.1289/ehp.00108301 PMC1638021

[31] Leaderer BP, Belanger K, Triche E, Holford T, Gold DR, et al (2002) Dust mite, cockroach, cat, and dog allergen concentrations in homes of asthmatic children in the northeastern United States: impact of socioeconomic factors and population density. Environ Health Perspect 110: 419–425. 11940461 10.1289/ehp.02110419 PMC1240806

[32] Muti D, Purohit A, Dazy A, Verot A, de Blay F (2012) Mouse (mus m1) and rat (rat n1) allergen levels in dust from private and public houses in Strasbourg, France are lower than houses in the U.S.A. Eur Ann Allergy Clin Immunol. 44: 93–95. 22768731

[33] Heederik D, von Mutius E (2012) Does diversity of environmental microbial exposure matter for the occurrence of allergy and asthma? J Allergy Clin Immunol 130: 44–50. 22502794 10.1016/j.jaci.2012.01.067

[34] Ege MJ, Mayer M, Normand AC, Genuneit J, Cookson WO, et al (2011) Exposure to environmental microorganisms and childhood asthma. N Engl J Med 364: 701–709. 21345099 10.1056/NEJMoa1007302

[35] Noss I, Wouters IM, Bezemer G, Metwali N, Sander I, et al (2010) Beta-(1,3)-glucan exposure assessment by passive airborne dust sampling and new sensitive immunoassays. Appl Environ Microbiol 76: 1158–1167. 20038709 10.1128/AEM.01486-09 PMC2820944

[36] Tischer C, Gehring U, Chen CM, Kerkhof M, Koppelman G, et al (2011) Respiratory health in children and indoor exposure to (1,3)-{beta}-D-glucan, EPS mould components, and endotoxin. Eur Respir J 37: 1050–1059. 20817706 10.1183/09031936.00091210

[37] Casas L, Tischer C, Wouters IM, Valkonen M, Gehring U, et al (2013) Endotoxin, extracellular polysaccharides, and beta(1–3)-glucan concentrations in dust and their determinants in four European birth cohorts: Results from the HITEA project. Indoor Air 23: 208–218. 23176390 10.1111/ina.12017

[38] Giovannangelo ME, Gehring U, Nordling E, Oldenwening M, van Rijswijk K, et al (2007) Levels and determinants of beta(1–>3)-glucans and fungal extracellular polysaccharides in house dust of (pre-)school children in three European countries. Environ Int 33: 9–16. 16859747 10.1016/j.envint.2006.06.018

